# A Case of Inversion of the Uterus, with Operation by White’s Method*This is the twelfth case of Dr. White’s series of successful restorations of the inverted uterus—*Ed. Buffalo M. & S. J.*

**Published:** 1876-01

**Authors:** E. C. Coxe

**Affiliations:** Buffalo, N. Y.


### A Case of Inversion of the Uterus, with Operation by White's Method.*

Reported by E. C. Coxe, M. D., Buffalo, N. Y.

Mrs. C., aged 25 years, is a native of Allegany county. She married when 18 years old, and had two confinements unmarked by any special incidents. On the 10th of June, 1875, she was brought to bed with her third child, and was attended by a practitioner who for some time had discontinued professional business.

Dr. W. S. Cottrell, the physician now in charge, gives the following history sf the case up to the date of the operation: "I was called to see Mrs. C. shortly after her delivery, the messenger stating that Dr. H. did not think that she would live five minutes. I immediately went to the house, and found the patient looking very badly; pale, in considerable pain; pulse 180, feeble in character. I asked the doctor if there had been flooding; he replied there had not, and said that he attributed the pallor and prostration to the morphine which he had administered, gr. ij., within twenty minutes. Shortly afterwards she vomited the morphine, and expressed herself as feeling some better. The doctor in charge then stated to me that the child was born with the cord around its neck, and that the placenta and uterus came down at the same time with the child, that he detached the placenta and returned the uterus. The women present say, however, that the child was born naturally, and that some time elapsed before the doctor 'took the after-birth,' and at the time there was some organ visible which they had never seen before in confinements.

* This is the twelfth case of Dr. White's series of successful restorations of the inverted uterus—*Ed. Buffalo M. & S. J.*

"The case was afterwards placed in my hands, and on visiting her four weeks after her delivery I found her feeble in health, taking but little food, and that liquid in character. Nevertheless, there was a large secretion of milk, on which the child throve.

"Dr. Shaw, of Erie county, being in the neighborhood, I determined to call him in consultation. Upon examination, a tumor was found lying in the vagina, which Dr. Shaw pronounced a uterine fibroid.

"This diagnosis was not satisfactory to me, so I sent for Dr. Crandall, of Andover, N. Y., to come in consultation, telling him to bring his instruments with him in case we should decide it was fibroid. On examining the patient, Dr. Crandall gave as his opinion that the tumor was an inverted uterus.

"At this date, August 24th, Mrs. C. being fearfully reduced, and subject to very frequent hæmorrhage, we decided that an operation for her relief should be attempted without delay."

Dr. Cottrell, at the request of the patient's husband, on the 9th of September wrote to Dr. White, asking him to operate. The doctor in reply named Sept. 22d as a convenient date, adding detailed directions in regard to preparing her for operation, *i. e.*, quinine and iron tonic, generous diet, perfect quiet, etc., laying *particular stress* upon the necessity of having the rectum thoroughly empty on the appointed day.

On Sept. 22d Dr. White arrived, and proceeded to make his preliminary examination, the following gentlemen being present and assisting: Drs. Cottrell, of Whitesville; Crandall, of Andover; Coxe, of Buffalo, and Mr. Langworthy, a student.

The patient being fully under the influence of ether, Dr. White made an attempt to examine by means of the sound in the bladder and the finger in the rectum; but the condition of the latter viscus, which, in spite of reiterated instruction, had been allowed to become loaded with hardened fæces, made this procedure impossible.

Upon introducing his finger into the vagina, the doctor was puzzled to find an object, which, in shape and consistence, differed greatly from the organ which he was in search of. After careful manipulation, he was enabled to draw forth a ball of cotton cloth saturated with horribly offensive putrid blood and discharge. Quite a quantity of black tarry fluid followed the withdrawal of the wad, filling the room with a stench truly infernal.

Dr. Cottrell explained that two weeks previous, Mrs. C. having a severe attack of flooding, Dr. Crandall had found it necessary to tampon the vagina, using for the purpose four pieces of cotton cloth. He had left directions for him, Dr. Cottrell, to remove them on the following day. This, as he thought, he had done, but events proved that a portion of the tampon had been left *in situ* for a *fortnight;* the uterus, by its agency, during the period being freely bathed in a highly septic fluid.

After thoroughly syringing the vagina, the doctor was enabled to make a perfectly satisfactory diagnosis by conjoined manipulation. By pushing up the tumor, the inverted os could be plainly felt through the walls of the abdomen, which were relaxed and thin. The uterus was not large, showing that the process of involution had not materilly been interfered with.

Introducing the right hand, armed with a large rectal bougie, into the vagina, while making counter-pressure with the left over the abdomen, partly by pressure, partly by manipulation, the walls of the uterus soon began to yield, and at the end of six minutes the doctor announced that the reduction was effected.

In this operation it was found unnecessary to use the "egg-beater" repositor. By comparing dates, we find that the uterus had been inverted for three months and twelve days.

The patient rallied nicely from the ether, the pulse was good, appearance quite cheerful. Feeling a good deal of uterine tenesmus, morphine was administered and followed by relief.

Particular directions were then given in regard to antiseptic injections. Carbolic water to be used freely, quinine to be given in full doses, and all pain subdued by opiates.

Subsequent to the operation, symptoms of septicæmia showed themselves. These, however, yielded promptly to appropriate treatment, and this danger averted, the patient began to make rapid progress towards a good recovery. On the fifteenth day after the operation, the menstrual flow appeared, continuing for two days. It was accompanied by the usual headache and slight abdominal pain, but no more than she was accustomed to experience at her "monthlies" before her pregnancy.

Eighteen days from date of operation the patient was able to sit up in a chair, ate with good appetite, and appeared so well that her attending physician discontinued his visits.—*Medical Record.*

